# The co-crystal 4,6-di­acetyl­resorcinol–1-amino­pyrene (2/1)

**DOI:** 10.1107/S2056989022005588

**Published:** 2022-05-31

**Authors:** Maryam Ali Magrashi, Elham Shafik Aazam

**Affiliations:** a Chemistry Department, Faculty of Science, King Abdulaziz University, Jeddah, 23622, Saudi Arabia; Moscow State University, Russia

**Keywords:** crystal structure, co-crystal, hydrogen bonds, stacking inter­actions, disorder

## Abstract

The structure of the title mol­ecular complex, C_16_H_11_N·2C_10_H_10_O_4_, at 150 K has been determined. The mol­ecules form stacks consisting of aggregates with disordered 1-amino­pyrene mol­ecule surrounded by two 4,6-di­acetyl­resorcinol mol­ecules. Neighbouring stacks are linked by hydrogen bonds between the amine H atoms of the 1-amino­pyrene mol­ecule with the adjacent carbonyl oxygen atom of the 4,6-di­acetyl­resorcinol mol­ecule.

## Abstract 2

In the title mol­ecular complex, the mol­ecules form stacks consisting of aggregates with disordered 1-amino­pyrene mol­ecule surrounded by two 4,6-di­acetyl­resorcinol mol­ecules. Neighbouring stacks are linked by hydrogen bonds between the amine H atoms of the 1-amino­pyrene mol­ecule with the adjacent carbonyl oxygen atom of the 4,6-di­acetyl­resorcinol mol­ecule.

## 1. Chemical context

Co-crystals are crystalline single phase materials made up of mol­ecules of two or more compounds. They are used in a variety of fields, including paper, textiles and the chemical, photographic, and electronic industries (Golbedaghi & Fausto, 2018 ▸). However, their main uses are centered in the pharmaceutical industry, where they have been gaining importance in recent years. 
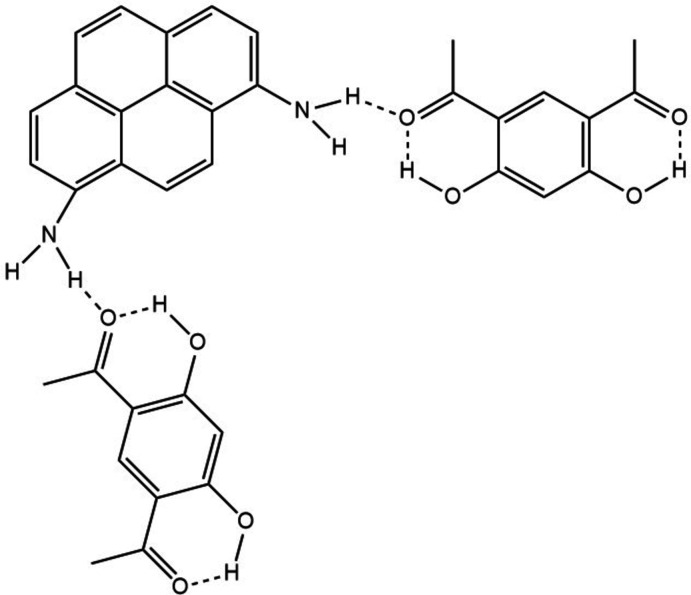


Schiff bases are the products of the condensation reaction of aldehydes or ketones with amines. They have multiple uses, for example as pigments and dyes, inter­mediates in organic synthesis, and as catalysts and polymer stabilizers. They also exhibit a broad range of biological activities. They play an important role in coordination chemistry as they readily form stable complexes with most transition metals (Aazam *et al.*, 2006 ▸, 2008 ▸, 2010 ▸; El-Attar & Aazam, 2021 ▸). In the process of the synthesis of such compounds with 4,6-di­acetyl­resorcinol and 1-amino­pyrene as the precursors, a new co-crystal, C_16_H_11_N·2C_10_H_10_O_4_, has been obtained.

## 2. Structural commentary

**Figure 1:**
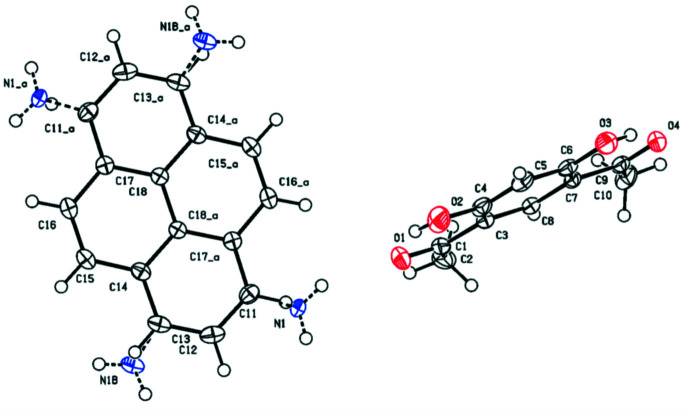
The asymmetric unit of the title compound with the atom labelling. Displacement ellipsoids are drawn at the 50% probability level.

The formula unit of the title compound consists of two 4,6-di­acetyl­resorcinol mol­ecules and one 1-amino­pyrene mol­ecule, which lies on an inversion center. Besides this, this mol­ecule is further disordered so that the amino N atom is distributed over four chemically equivalent positions, at the C11 and C13 atoms, with the occupancies of 0.428 (2) and 0.072 (2) for N1 and N1*B*, respectively (Fig. 1 ▸).

In the 4,6-di­acetyl­resorcinol mol­ecule, the hy­droxy groups form intra­molecular hydrogen bonds with the oxygen atoms of neighbouring acetyl groups, generating *S*(6) rings (Table 1 ▸).

## 3. Supra­molecular features

**Figure 2:**
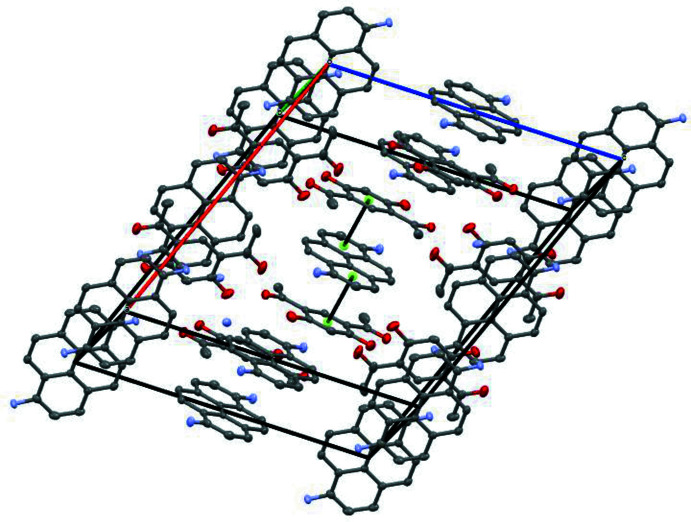
A view of the crystal packing showing the π–π stacking inter­actions.

**Figure 3:**
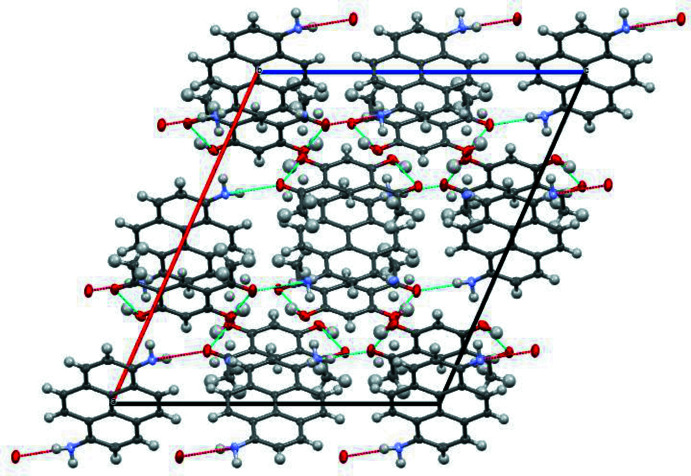
The crystal packing of the title compound viewed along the *b* axis, showing the N—H⋯O hydrogen bonds.

In the crystal, the mol­ecules form centrosymmetric aggregates with two mol­ecules of 4,6-di­acetyl­resorcinol positioned on both sides of the 1-amino­pyrene mol­ecule (Fig. 2 ▸). The mean planes of the aromatic rings of the 4,6-di­acetyl­resorcinol mol­ecules are inclined at 2.91 (10)° to the mean plane of the tetra­cyclic core of the 1-amino­pyrene mol­ecule. A short inter­centroid separation *Cg*1⋯*Cg*2 of 3.492 (1) Å is observed in this aggregate, with *Cg*1 being the centroid of C3–C8 ring of di­acetyl­resorcinol and *Cg*2 the centroid of one of the amino­pyrene rings, C11–C18. These aggregates are packed into stacks by π–π stacking inter­actions between 4,6-di­acetyl­resorcinol mol­ecules. Neighbouring stacks are linked by hydrogen bonds between the amino H atom of the 1-amino­pyrene mol­ecule with the adjacent carbonyl oxygen atom of the 4,6-di­acetyl­resorcinol mol­ecule, thus forming a three-dimensional network (Fig. 3 ▸).

## 4. Database survey

A search of the Cambridge Structural Database (CSD, Version 5.42; May 2021; Groom *et al.*, 2016 ▸) gave the structures of the individual components. In the structure of 4,6-di­acetyl­resorcinol (Kokila *et al.*, 1992 ▸, refcode VOXPED) the mol­ecule is almost planar, with the oxygen atoms of the acetyl groups deviating by 0.074 (1) and 0.072 (2) Å from the mean plane of the benzene ring. There are intra­molecular hydrogen bonds between the oxygen atoms of the acetyl groups and the hy­droxy hydrogen atoms. A search for 1-amino­pyrene gave two hits for co-crystals composed of 1-amino­pyrene mol­ecules with either 7,7′,8,8′-tetra­cyano­quinodi­methane or 3,5-di­nitro­benzoic acid and showed that the NH_2_ groups can act as H-donors in the inter­molecular hydrogen-bonding inter­actions, as in the title compound. (Mandal *et al.*, 2020 ▸, refcode BOYQOY; Mandal *et al.*, 2019 ▸, refcode LORBOM).

## 5. Synthesis and crystallization

A solution of 1-amino­pyrene (1 mmol, 0.233 g) dissolved in 10 ml of ethanol was added dropwise to 4,6-di­acetyl­resorcinol (DAR) (0.5 mmol, 0.097 g) dissolved in 10 ml of ethanol, 3 drops of acetic acid were added, and the mixture was stirred for 15 min at room temperature and then for about 3 h under reflux. Yellow fiber-like crystals of the Schiff base ligand were separated. They were filtered off and washed with 4 ml of ethanol, weight = 0.021 g, yield = 7.12%, m.p. = 523 K, *m*/*z* = 592.7 (C_42_H_28_N_2_O_2_). The filtrate was left overnight upon which dark-brown rectangular co-crystals were formed, weight = 0.04 g, yield = 19.5%, m.p. = 418 K, *m*/*z* = 605.62 (C_16_H_11_N·2C_10_H_10_O_4_). ^1^H NMR (800 MHz, DMSO-*d*_6_) δ 12.75 (*s*, *br*, –OH), 8.406 (*s*, 2H, DAR), 8.251 (*d*, 1H), 7.992 (*d*, 2H), 7.992 (*d*, 1H), 7.958 (*d*, 1H), 87.915 (*d*, 2H), 7.880 (*m*, 1H), 7.367 (*d*, 1H), 6.392 (*s*, 2H, DAR), 6.314 (*s*, *br*, NH2, 2H), 2.661 (*s*, Me, 12H).

## 6. Refinement

Crystal data, data collection and structure refinement details are summarized in Table 2 ▸. The C—N bond distances for the disordered N atom were restrained to be similar. The minor occupancy N1*B* atom was constrained to have the same ADPs as the C atom to which it is bonded. N—H bond distances were restrained to a target value of 0.88 (2) Å, and the H—N—H and C—N—H bond angles were restrained to be similar to each other. Subject to these conditions the occupancy rates refined to 0.428 (2) and 0.072 (2). O-bound H atoms were refined with *U*_iso_(H) = 1.5*U*_eq_(O). C-bound H atoms were positioned geometrically (C—H = 0.9–0.98 Å) and refined as riding on their parent atoms with *U*_iso_(H) = 1.2–1.5*U*_eq_(C).

## Supplementary Material

Crystal structure: contains datablock(s) I, global. DOI: 10.1107/S2056989022005588/yk2169sup1.cif

Structure factors: contains datablock(s) I. DOI: 10.1107/S2056989022005588/yk2169Isup2.hkl

Supporting information file. DOI: 10.1107/S2056989022005588/yk2169Isup3.cml

CCDC reference: 2174187

Additional supporting information:  crystallographic information; 3D view; checkCIF report

## Figures and Tables

**Table 1:** Hydrogen-bond geometry (Å, °)

*D*—H⋯*A*	*D*—H	H⋯*A*	*D*⋯*A*	*D*—H⋯*A*
O2—H2⋯O1	0.85 (3)	1.80 (3)	2.545 (2)	146 (2)
O3—H3⋯O4	0.84 (2)	1.80 (2)	2.542 (2)	147 (2)
N1—H1*A*⋯O3^i^	0.88 (1)	2.30 (2)	3.131 (3)	156 (4)
N1—H1*B*⋯O1	0.88 (1)	2.16 (2)	2.966 (3)	154 (3)
N1*B*—H1*C*⋯O2^ii^	0.88 (1)	2.05 (6)	2.902 (18)	162 (17)
N1*B*—H1*D*⋯O4^iii^	0.88 (1)	1.89 (6)	2.731 (18)	159 (14)

Symmetry codes: (i) 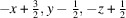 ; (ii) 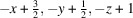 ; (iii) 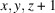 .

**Table 2:** Experimental details

Crystal data	
Chemical formula	C_16_H_11_N·2C_10_H_10_O_4_
*M*_r_	605.62
Crystal system, space group	Monoclinic, *C*2/*c*
Temperature (K)	150
*a*, *b*, *c* (Å)	18.7222 (14), 9.7870 (6), 16.9398 (15)
β (°)	113.758 (4)
*V* (Å^3^)	2840.9 (4)
*Z*	4
Radiation type	Mo *K*α
μ (mm^−1^)	0.10
Crystal size (mm)	0.45 × 0.26 × 0.22

Data collection	
Diffractometer	Bruker AXS D8 Quest diffractometer with PhotonII charge-integrating pixel array detector (CPAD)
Absorption correction	Multi-scan (*SADABS*; Krause *et al.*, 2015 ▸)
*T*_min_, *T*_max_	0.673, 0.747
No. of measured, independent and observed [*I* > 2σ(*I*)] reflections	33773, 4316, 3741
*R*_int_	0.046
(sin θ/λ)_max_ (Å^−1^)	0.714

Refinement	
*R*[*F* ^2^ > 2σ(*F* ^2^)], *wR*(*F* ^2^), *S*	0.061, 0.153, 1.15
No. of reflections	4316
No. of parameters	231
No. of restraints	13
H-atom treatment	H atoms treated by a mixture of independent and constrained refinement
Δρ_max_, Δρ_min_ (e Å^−3^)	0.35, −0.26

Computer programs: *APEX4* and *SAINT* (Bruker, 2021 ▸), *SHELXT* (Sheldrick, 2015*a* ▸), *SHELXL2019/2* (Sheldrick, 2015*b* ▸), *ShelXle* (Hübschle *et al.*, 2011 ▸) and *publCIF* (Westrip, 2010 ▸).

## supplementary crystallographic information

### Crystal data

**Table:**

C_16_H_11_N·2C_10_H_10_O_4_	*F*(000) = 1272
*M**_r_* = 605.62	*D*_x_ = 1.416 Mg m^−^^3^
Monoclinic, *C*2/*c*	Mo *K*α radiation, λ = 0.71073 Å
*a* = 18.7222 (14) Å	Cell parameters from 9850 reflections
*b* = 9.7870 (6) Å	θ = 2.4–33.2°
*c* = 16.9398 (15) Å	µ = 0.10 mm^−^^1^
β = 113.758 (4)°	*T* = 150 K
*V* = 2840.9 (4) Å^3^	Block, yellow
*Z* = 4	0.45 × 0.26 × 0.22 mm

### Data collection

**Table:**

Bruker AXS D8 Quest diffractometer with PhotonII charge-integrating pixel array detector (CPAD)	4316 independent reflections
Radiation source: fine focus sealed tube X-ray source	3741 reflections with *I* > 2σ(*I*)
Triumph curved graphite crystal monochromator	*R*_int_ = 0.046
Detector resolution: 7.4074 pixels mm^-1^	θ_max_ = 30.5°, θ_min_ = 2.5°
ω and phi scans	*h* = −26→26
Absorption correction: multi-scan (SADABS; Krause *et al.*, 2015)	*k* = −13→13
*T*_min_ = 0.673, *T*_max_ = 0.747	*l* = −24→24
33773 measured reflections	

### Refinement

**Table:**

Refinement on *F*^2^	Primary atom site location: dual
Least-squares matrix: full	Secondary atom site location: difference Fourier map
*R*[*F*^2^ > 2σ(*F*^2^)] = 0.061	Hydrogen site location: mixed
*wR*(*F*^2^) = 0.153	H atoms treated by a mixture of independent and constrained refinement
*S* = 1.15	*w* = 1/[σ^2^(*F*_o_^2^) + (0.0356*P*)^2^ + 4.9256*P*] where *P* = (*F*_o_^2^ + 2*F*_c_^2^)/3
4316 reflections	(Δ/σ)_max_ < 0.001
231 parameters	Δρ_max_ = 0.35 e Å^−^^3^
13 restraints	Δρ_min_ = −0.26 e Å^−^^3^

### Special details

**Table:**

Geometry. All esds (except the esd in the dihedral angle between two l.s. planes) are estimated using the full covariance matrix. The cell esds are taken into account individually in the estimation of esds in distances, angles and torsion angles; correlations between esds in cell parameters are only used when they are defined by crystal symmetry. An approximate (isotropic) treatment of cell esds is used for estimating esds involving l.s. planes.
Refinement. The single nitrogen atom is disordered over four chemically equivalent positions (each two are also crystallographically equivalent, by inversion). The C-N bond distances were restrained to be similar. The minor N atom was constrained to have the same ADP as the C atom it is bonded to. The N-H bond distances were restrained to a target value of 0.88 (2) Angstrom, and the H-N-H and C-N-H bond angles were each restrained to be similar to each other. Subject to these conditions the occupancy rates refined to two times 0.428 (2) and two times 0.072 (2).

### Fractional atomic coordinates and isotropic or equivalent isotropic displacement parameters (Å^2^)

**Table:**

	*x*	*y*	*z*	*U*_iso_*/*U*_eq_	Occ. (<1)
O1	0.65970 (9)	0.27504 (17)	0.27887 (8)	0.0435 (4)	
O2	0.73863 (8)	0.46240 (16)	0.24707 (9)	0.0406 (3)	
H2	0.7158 (15)	0.422 (3)	0.2746 (11)	0.061*	
O3	0.73548 (7)	0.49314 (13)	−0.02964 (9)	0.0318 (3)	
H3	0.7141 (13)	0.459 (2)	−0.0788 (16)	0.048*	
O4	0.65513 (7)	0.32200 (14)	−0.14377 (8)	0.0333 (3)	
N1	0.63242 (19)	0.2052 (3)	0.43483 (19)	0.0232 (6)	0.428 (2)
H1A	0.6785 (13)	0.167 (4)	0.462 (2)	0.028*	0.428 (2)
H1B	0.636 (3)	0.252 (3)	0.3924 (19)	0.028*	0.428 (2)
N1B	0.6296 (10)	0.2240 (18)	0.6962 (10)	0.0255 (3)	0.072 (2)
H1C	0.676 (5)	0.184 (17)	0.716 (6)	0.031*	0.072 (2)
H1D	0.625 (10)	0.261 (10)	0.741 (4)	0.031*	0.072 (2)
C1	0.63682 (10)	0.23098 (19)	0.20409 (11)	0.0303 (4)	
C2	0.58258 (12)	0.1108 (2)	0.17814 (13)	0.0392 (4)	
H2A	0.535960	0.134104	0.126697	0.059*	
H2B	0.567315	0.086926	0.225370	0.059*	
H2C	0.609006	0.032779	0.165398	0.059*	
C3	0.66261 (9)	0.29590 (16)	0.14205 (9)	0.0232 (3)	
C4	0.71264 (9)	0.41203 (17)	0.16646 (10)	0.0264 (3)	
C5	0.73613 (9)	0.47613 (17)	0.10833 (11)	0.0265 (3)	
H5	0.769710	0.553232	0.125827	0.032*	
C6	0.71078 (8)	0.42813 (15)	0.02447 (10)	0.0228 (3)	
C7	0.66054 (8)	0.31225 (15)	−0.00255 (9)	0.0194 (3)	
C8	0.63794 (8)	0.24981 (15)	0.05741 (9)	0.0206 (3)	
H8	0.604356	0.172691	0.039992	0.025*	
C9	0.63396 (9)	0.26350 (17)	−0.09192 (10)	0.0242 (3)	
C10	0.58093 (10)	0.1419 (2)	−0.12108 (12)	0.0339 (4)	
H10A	0.568838	0.123625	−0.182006	0.051*	
H10B	0.532509	0.160674	−0.113824	0.051*	
H10C	0.606809	0.062098	−0.086448	0.051*	
C11	0.60624 (9)	0.27031 (15)	0.48402 (10)	0.0237 (3)	
H11	0.624879	0.227360	0.445699	0.028*	0.572 (2)
C12	0.63162 (9)	0.22506 (16)	0.56916 (11)	0.0283 (3)	
H12	0.666448	0.149723	0.587700	0.034*	
C13	0.60710 (9)	0.28760 (16)	0.62685 (10)	0.0255 (3)	
H13	0.625984	0.256168	0.684761	0.031*	0.928 (2)
C14	0.55427 (8)	0.39772 (15)	0.60056 (9)	0.0207 (3)	
C15	0.52761 (9)	0.46518 (16)	0.65871 (9)	0.0240 (3)	
H15	0.546435	0.435794	0.717036	0.029*	
C16	0.47590 (9)	0.57043 (16)	0.63217 (9)	0.0232 (3)	
H16	0.458483	0.611875	0.671966	0.028*	
C17	0.44718 (8)	0.61998 (15)	0.54519 (9)	0.0196 (3)	
C18	0.47321 (8)	0.55597 (14)	0.48616 (9)	0.0174 (3)	

### Atomic displacement parameters (Å^2^)

**Table:**

	*U*^11^	*U*^22^	*U*^33^	*U*^12^	*U*^13^	*U*^23^
O1	0.0494 (8)	0.0610 (10)	0.0232 (6)	0.0200 (7)	0.0178 (6)	0.0096 (6)
O2	0.0380 (7)	0.0502 (9)	0.0258 (6)	0.0040 (6)	0.0048 (5)	−0.0143 (6)
O3	0.0326 (6)	0.0283 (6)	0.0415 (7)	−0.0002 (5)	0.0223 (6)	0.0058 (5)
O4	0.0363 (6)	0.0425 (7)	0.0241 (6)	0.0130 (6)	0.0153 (5)	0.0058 (5)
N1	0.0294 (15)	0.0217 (14)	0.0224 (14)	0.0077 (11)	0.0147 (12)	0.0002 (11)
N1B	0.0237 (7)	0.0230 (7)	0.0254 (7)	−0.0008 (5)	0.0052 (6)	0.0077 (6)
C1	0.0299 (8)	0.0382 (9)	0.0254 (7)	0.0158 (7)	0.0137 (6)	0.0107 (7)
C2	0.0421 (10)	0.0399 (10)	0.0448 (10)	0.0109 (8)	0.0270 (9)	0.0204 (8)
C3	0.0221 (6)	0.0265 (7)	0.0211 (6)	0.0074 (5)	0.0087 (5)	0.0025 (5)
C4	0.0207 (7)	0.0296 (8)	0.0242 (7)	0.0067 (6)	0.0039 (5)	−0.0056 (6)
C5	0.0188 (6)	0.0233 (7)	0.0346 (8)	−0.0001 (5)	0.0078 (6)	−0.0063 (6)
C6	0.0176 (6)	0.0205 (6)	0.0314 (7)	0.0043 (5)	0.0111 (5)	0.0021 (6)
C7	0.0170 (6)	0.0205 (6)	0.0202 (6)	0.0038 (5)	0.0071 (5)	−0.0006 (5)
C8	0.0189 (6)	0.0194 (6)	0.0236 (7)	0.0027 (5)	0.0086 (5)	0.0001 (5)
C9	0.0211 (6)	0.0274 (7)	0.0221 (7)	0.0090 (5)	0.0067 (5)	−0.0008 (6)
C10	0.0278 (8)	0.0369 (9)	0.0321 (8)	0.0013 (7)	0.0067 (6)	−0.0151 (7)
C11	0.0222 (6)	0.0194 (6)	0.0305 (7)	−0.0009 (5)	0.0117 (6)	−0.0020 (6)
C12	0.0236 (7)	0.0205 (7)	0.0372 (9)	0.0021 (5)	0.0084 (6)	0.0054 (6)
C13	0.0237 (7)	0.0230 (7)	0.0254 (7)	−0.0008 (5)	0.0052 (6)	0.0077 (6)
C14	0.0184 (6)	0.0217 (7)	0.0205 (6)	−0.0035 (5)	0.0065 (5)	0.0032 (5)
C15	0.0240 (7)	0.0291 (8)	0.0178 (6)	−0.0041 (6)	0.0074 (5)	0.0021 (5)
C16	0.0241 (7)	0.0275 (7)	0.0190 (6)	−0.0045 (6)	0.0100 (5)	−0.0016 (5)
C17	0.0188 (6)	0.0187 (6)	0.0213 (6)	−0.0036 (5)	0.0081 (5)	−0.0010 (5)
C18	0.0157 (6)	0.0171 (6)	0.0186 (6)	−0.0036 (5)	0.0060 (5)	−0.0002 (5)

### Geometric parameters (Å, º)

**Table:**

O1—C1	1.240 (2)	C7—C8	1.389 (2)
O2—C4	1.3450 (19)	C7—C9	1.470 (2)
O2—H2	0.84 (3)	C8—H8	0.9500
O3—C6	1.3418 (19)	C9—C10	1.500 (2)
O3—H3	0.83 (3)	C10—H10A	0.9800
O4—C9	1.240 (2)	C10—H10B	0.9800
N1—C11	1.292 (3)	C10—H10C	0.9800
N1—H1A	0.882 (10)	C11—C12	1.396 (2)
N1—H1B	0.875 (10)	C11—C17^i^	1.414 (2)
N1B—C13	1.243 (14)	C11—H11	0.9500
N1B—H1C	0.882 (10)	C12—C13	1.379 (2)
N1B—H1D	0.880 (10)	C12—H12	0.9500
C1—C3	1.467 (2)	C13—C14	1.408 (2)
C1—C2	1.500 (3)	C13—H13	0.9500
C2—H2A	0.9800	C14—C18^i^	1.4210 (19)
C2—H2B	0.9800	C14—C15	1.432 (2)
C2—H2C	0.9800	C15—C16	1.360 (2)
C3—C8	1.392 (2)	C15—H15	0.9500
C3—C4	1.424 (2)	C16—C17	1.434 (2)
C4—C5	1.380 (2)	C16—H16	0.9500
C5—C6	1.386 (2)	C17—C18	1.4220 (19)
C5—H5	0.9500	C18—C18^i^	1.431 (3)
C6—C7	1.426 (2)		
			
C4—O2—H2	109.5	O4—C9—C10	119.42 (15)
C6—O3—H3	109.5	C7—C9—C10	120.08 (14)
C11—N1—H1A	114 (2)	C9—C10—H10A	109.5
C11—N1—H1B	116 (2)	C9—C10—H10B	109.5
H1A—N1—H1B	106 (4)	H10A—C10—H10B	109.5
C13—N1B—H1C	120 (3)	C9—C10—H10C	109.5
C13—N1B—H1D	120 (3)	H10A—C10—H10C	109.5
H1C—N1B—H1D	106 (5)	H10B—C10—H10C	109.5
O1—C1—C3	120.23 (18)	N1—C11—C12	116.75 (19)
O1—C1—C2	119.14 (16)	N1—C11—C17^i^	123.18 (19)
C3—C1—C2	120.63 (15)	C12—C11—C17^i^	120.05 (14)
C1—C2—H2A	109.5	C12—C11—H11	120.0
C1—C2—H2B	109.5	C17^i^—C11—H11	120.0
H2A—C2—H2B	109.5	C13—C12—C11	121.28 (14)
C1—C2—H2C	109.5	C13—C12—H12	119.4
H2A—C2—H2C	109.5	C11—C12—H12	119.4
H2B—C2—H2C	109.5	N1B—C13—C12	111.3 (10)
C8—C3—C4	117.66 (14)	N1B—C13—C14	127.6 (10)
C8—C3—C1	121.95 (15)	C12—C13—C14	120.49 (14)
C4—C3—C1	120.37 (15)	C12—C13—H13	119.8
O2—C4—C5	117.95 (16)	C14—C13—H13	119.8
O2—C4—C3	120.93 (16)	C13—C14—C18^i^	119.12 (14)
C5—C4—C3	121.12 (14)	C13—C14—C15	122.02 (14)
C4—C5—C6	120.08 (15)	C18^i^—C14—C15	118.86 (13)
C4—C5—H5	120.0	C16—C15—C14	121.37 (14)
C6—C5—H5	120.0	C16—C15—H15	119.3
O3—C6—C5	118.00 (15)	C14—C15—H15	119.3
O3—C6—C7	121.48 (14)	C15—C16—C17	121.20 (14)
C5—C6—C7	120.52 (14)	C15—C16—H16	119.4
C8—C7—C6	118.10 (13)	C17—C16—H16	119.4
C8—C7—C9	122.26 (14)	C11^i^—C17—C18	118.88 (13)
C6—C7—C9	119.65 (14)	C11^i^—C17—C16	122.40 (14)
C7—C8—C3	122.53 (14)	C18—C17—C16	118.72 (13)
C7—C8—H8	118.7	C14^i^—C18—C17	120.17 (13)
C3—C8—H8	118.7	C14^i^—C18—C18^i^	119.80 (16)
O4—C9—C7	120.50 (15)	C17—C18—C18^i^	120.04 (16)
			
O1—C1—C3—C8	−179.71 (15)	C6—C7—C9—O4	0.3 (2)
C2—C1—C3—C8	0.3 (2)	C8—C7—C9—C10	−0.3 (2)
O1—C1—C3—C4	−1.5 (2)	C6—C7—C9—C10	−179.83 (13)
C2—C1—C3—C4	178.49 (14)	N1—C11—C12—C13	180.0 (2)
C8—C3—C4—O2	179.86 (14)	C17^i^—C11—C12—C13	−1.6 (2)
C1—C3—C4—O2	1.6 (2)	C11—C12—C13—N1B	173.1 (9)
C8—C3—C4—C5	−0.3 (2)	C11—C12—C13—C14	1.3 (2)
C1—C3—C4—C5	−178.61 (14)	N1B—C13—C14—C18^i^	−170.7 (11)
O2—C4—C5—C6	−179.90 (14)	C12—C13—C14—C18^i^	−0.3 (2)
C3—C4—C5—C6	0.3 (2)	N1B—C13—C14—C15	9.6 (11)
C4—C5—C6—O3	−179.61 (14)	C12—C13—C14—C15	−179.94 (14)
C4—C5—C6—C7	−0.2 (2)	C13—C14—C15—C16	−179.26 (14)
O3—C6—C7—C8	179.53 (13)	C18^i^—C14—C15—C16	1.1 (2)
C5—C6—C7—C8	0.1 (2)	C14—C15—C16—C17	−1.3 (2)
O3—C6—C7—C9	−1.0 (2)	C15—C16—C17—C11^i^	−179.63 (14)
C5—C6—C7—C9	179.64 (13)	C15—C16—C17—C18	0.6 (2)
C6—C7—C8—C3	−0.2 (2)	C11^i^—C17—C18—C14^i^	0.1 (2)
C9—C7—C8—C3	−179.68 (13)	C16—C17—C18—C14^i^	179.82 (13)
C4—C3—C8—C7	0.3 (2)	C11^i^—C17—C18—C18^i^	−179.52 (15)
C1—C3—C8—C7	178.53 (13)	C16—C17—C18—C18^i^	0.2 (2)
C8—C7—C9—O4	179.78 (14)		

Symmetry code: (i) −*x*+1, −*y*+1, −*z*+1.

### Hydrogen-bond geometry (Å, º)

**Table:**

*D*—H···*A*	*D*—H	H···*A*	*D*···*A*	*D*—H···*A*
O2—H2···O1	0.85 (3)	1.80 (3)	2.545 (2)	146 (2)
O3—H3···O4	0.84 (2)	1.80 (2)	2.542 (2)	147 (2)
N1—H1*A*···O3^ii^	0.88 (1)	2.30 (2)	3.131 (3)	156 (4)
N1—H1*B*···O1	0.88 (1)	2.16 (2)	2.966 (3)	154 (3)
N1*B*—H1*C*···O2^iii^	0.88 (1)	2.05 (6)	2.902 (18)	162 (17)
N1*B*—H1*D*···O4^iv^	0.88 (1)	1.89 (6)	2.731 (18)	159 (14)

Symmetry codes: (ii) −*x*+3/2, *y*−1/2, −*z*+1/2; (iii) −*x*+3/2, −*y*+1/2, −*z*+1; (iv) *x*, *y*, *z*+1.
